# Effects of Cannabidiol (CBD) on Doxorubicin-Induced Anxiety and Depression-like Behaviors and mRNA Expression of Inflammatory Markers in Rats

**DOI:** 10.3390/brainsci14100999

**Published:** 2024-09-30

**Authors:** Bristi Poudel, Brent M. Bany, Dale Buchanan Hales, Joseph L. Cheatwood

**Affiliations:** Department of Biomedical Sciences, Southern Illinois University School of Medicine, Carbondale, IL 62901, USA; bristi.poudel@siu.edu (B.P.);

**Keywords:** chemo-brain, cannabidiol, doxorubicin, cognitive deficits, neuroinflammation

## Abstract

**Background**: Post-treatment side effects of chemotherapy can include cognitive deficits commonly known as Chemo-brain. The treatment of patients with Doxorubicin (DOX), one of the most widely used chemotherapeutic drugs in the treatment of cancer, can induce depression, anxiety, and impaired cognitive function. Cannabidiol (CBD) is a non-psychoactive component of *Cannabis sativa* that has been identified as a possible therapeutic agent against many neurodegenerative disorders, including traumatic brain injury, spinal cord injury, Tau-protein-induced neurodegeneration, and neuropathic pain. Therefore, this study aimed to assess whether oral CBD administration could reduce DOX-induced anxiety and depression-like behaviors and alter the expression of mRNA associated with neuroinflammation. **Methods**: Female Long Evans Hooded rats received intraperitoneal injections of DOX (6 mg/kg) or the vehicle (0.9% saline) once a week for four weeks, followed by oral administration of CBD (10 mg/kg) three times a week for the same period. **Results**: CBD was significantly protective against DOX-induced anxiety and depression-like behaviors, as measured by several behavioral tests. Furthermore, CBD improved DOX-induced alterations in the gene expression of biomarkers of neuroinflammation in the hippocampus and prefrontal cortex. **Conclusions**: This provides insights into future studies on possible mechanisms by which DOX-induced cognitive dysfunction could be alleviated by CBD.

## 1. Introduction

Chemotherapy regimens are used to treat various types of cancers and can lengthen survival for cancer patients [1]. Despite the effectiveness of chemotherapeutic agents, they often produce neurological side effects [1]. Almost all categories of chemotherapeutic agents can negatively affect memory and cognition [1,2]. Up to 75% of patients develop cognitive deficits after chemotherapy, and deficits remain after treatment ends in 34% of patients [3,4]. Cognitive deficits arising from chemotherapy treatment are commonly called “Chemo-brain” (CB). CB results in structural and functional brain changes following chemotherapy and is a potentially enfeebling condition that includes deficits in attention, visual and verbal memory, executive function, information processing, and short-term memory [2,5,6]. Doxorubicin (DOX) is one of the most widely used chemotherapeutic drugs in cancer treatment [7]. DOX disrupts replication in cancer cells via DNA intercalation and topoisomerase II inhibition [8] and produces reactive superoxide radical anions [9]. DOX can acutely elevate the levels of inflammatory cytokines, leading to glial cell activation [10]. Activated glial cells further cause the tremendous release of pro-inflammatory cytokines and reactive oxygen species (ROS) in the brain, which is one of the hypothesized mechanisms by which DOX treatment induces cognitive deficits [10,11,12]. Others have reported DOX-induced oxidative stress, inflammation, mitochondrial dysfunction, reduced neurogenesis, alterations in neurotransmitter levels, and dysregulation of apoptosis [4]. In human patients, treatment with DOX has been tied to neurological disorders like depression, anxiety, and reduced cognitive function [5,10].

Over the last decade, there has been a growing interest in investigating the potential therapeutic uses of cannabinoid compounds. The cannabinoid system relies on the G-protein-coupled receptors CB1 (Cannabinoid Receptor 1) and CB2 (Cannabinoid Receptor 2), which are highly expressed in both neurons and glia [13,14]. In central nervous system (CNS) tissue, CB2 expression is upregulated by microglia during neuroinflammatory events [14]. The CNR1 gene encodes CBR1, and CBR2 is encoded by the CNR2 gene, consisting of 360 amino acids in humans [15]. They belong to the member of the class G protein-coupled receptor family with glycosylated extracellular amino-terminal (N-terminal) and an intracellular carboxyl-terminal (C-term) [16]. There are over 120 known phytocannabinoids, of which Tetrahydrocannabinol (THC) and Cannabidiol (CBD) are currently the most studied to date [17,18,19,20]. CBD is one of the most abundant phytocannabinoids, comprising 40% of the plant extract [21]. It is commonly found in hemp plants and possesses a unique ability to antagonize CBR1 [22]. The antagonistic effect of CBD on CBR1 modulates anxiety, hunger, and sedation in both humans and rodents despite its low binding affinity for cannabinoid receptors [22]. CBD has been identified as a possible therapeutic agent for use in treating many neurological disorders, including traumatic brain injury [17], spinal cord injury [23], Tau-protein-induced neurodegeneration [23], and neuropathic pain [19,24]. However, there is a paucity of data regarding the potential benefits of CBD in a rodent Chemo-brain model.

Therefore, in the current experiments, we aimed to assess the effects of CBD administration in DOX-induced anxiety and depression using behavioral tests in a rodent model and elucidate its effects on the expression of mRNA of interest in the hippocampus and cerebral cortex.

## 2. Materials and Methods

### 2.1. Animals and Experimental Design

Our study was carried out in accordance with the U.S National Institutes of Health Guide for the Care and Use of Laboratory Animals. All procedures were approved by the Southern Illinois University Institutional Animal Care and Use Committee (IACUC protocol 22-005 approval on 2 August 2022).

Eight-week-old female Long Evans Hooded rats (Charles River Laboratories, Wilmington, MA, USA) were maintained on a 12:12 light–dark cycle and allowed ad libitum access to food and water. Rats were single-housed in the animal facility vivarium to accurately measure water and food intake, allowing for the effective assessment of the Sucrose Preference Test and the effects of appetite. Rats were handled daily for one week before experiments began to allow them to acclimate to their surroundings and to the investigators. Rats were then randomly allocated to one of four groups. Animals were assigned to experimental groups using a random number table, ensuring that allocation was unbiased.

**Figure 1:**
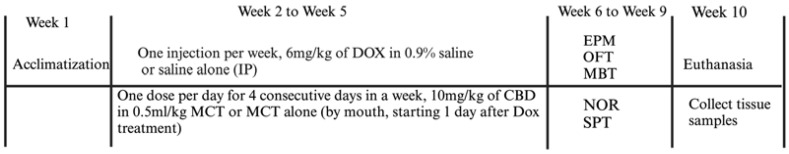
Experimental timeline. After acclimatization, rats were injected intraperitoneally with DOX (6 mg/kg) or the vehicle. Beginning the day after injection, rats received either CBD (10 mg/kg in MCT oil; 4 consecutive days each week; oral route) or MCT oil alone (0.5 mL/kg) for 4 consecutive weeks. Behavioral tests were conducted serially over 4 weeks (in the order shown from top to bottom), beginning one week after experimental treatments concluded. At the end of the study, rats were euthanized, and brain tissue samples were collected for qPCR assay at week 10. DOX—doxorubicin; CBD—Cannabidiol; MCT—Medium-Chain Triglyceride; IP—intraperitoneal; EPM—Elevated Plus Maze; OFT—Open Field Test; NOR—Novel Object Recognition; MBT—Marble Burying Test; SPT—Sucrose Preference Test.

Group 1 (Control, *n* = 4) received an intraperitoneal (IP) injection of 0.9% sterile saline and oral administration of 0.5 mL/kg MCTs (Medium-Chain Triglycerides; Garden of Life LLC, Palm Beach Gardens, FL, USA). Group 2 (DOX, *n* = 4) received an IP injection of DOX (6 mg/kg in 0.9% saline; Thermo Fisher Scientific, Waltham, MA, USA) plus oral administration of MCTs (0.5 mL/kg). Group 3 (DOX + CBD, *n* = 4) received an IP injection of DOX (6 mg/kg in 0.9% saline) and oral administration of CBD (10 mg/kg in a total volume of 0.5 mL/kg MCT; Industrial Hemp, Farms Smith Road, Denver CO). Group 4 (CBD, *n* = 4) received an IP injection of 0.9% sterile saline and oral administration of CBD isolate (10 mg/kg in a volume of 0.5 mL/kg MCT). The dose and treatment duration were chosen based on previous research by others that demonstrated DOX-induced cognitive effects [7,24,25] and/or neuroprotective efficacy of CBD in rodent models of anxiety [26,27], depression [17,28], and neuropathic pain [19,28]. Body weight and food consumed by each animal were recorded throughout the experiment, and the drug dose was adjusted accordingly. The experimenter who conducted behavioral testing was blind to the treatment groups. This blinding process was crucial to ensure unbiased analysis of outcomes. The experimental timeline is shown in Figure 1.

### 2.2. Behavioral Assessment

All behavioral tests were performed from week 6 to week 9 in the order presented in the sections below (i.e., EPM, OFT, MBT, NOR, SPT; also shown in Figure 1 from top to bottom).

#### 2.2.1. Elevated Plus Maze (EPM)

The EPM task was used to evaluate anxiety-like behaviors, as performed previously by others [29]. For this, each rat was placed in the center of the apparatus, which consisted of two open arms (45 cm) and two closed arms (45 cm), and was allowed to explore freely for 5 min. Videos of each trial were recorded by a camera positioned above the apparatus, and all videos were analyzed using ANY-maze video tracking software, Version 7.3 (Wood Dale, IL, USA), monitoring the distance traveled in the open arms and closed arms, as well as the time spent in open arms and closed arms during the test. After each test, the rat was returned to its home cage, and the maze was cleaned with 70% ethanol before the next trial.

#### 2.2.2. Open Field Test (OFT)

The OFT was performed to evaluate rats’ anxiety and exploratory and locomotory behavior [29]. The test was performed in an open field box designed for rats (45 cm height, 66 cm length, 66 cm width). The rats were individually placed at the arena’s center and allowed to explore for five minutes freely. Behaviors were recorded using a video camera mounted on the ceiling and analyzed with ANY-maze video tracking software. Time spent in the center, time spent in corners, and total distance traveled in each zone were quantified for comparison across groups.

#### 2.2.3. Marble Burying Test (MBT)

The MBT was performed to assess anxiety-like behaviors as performed previously by others [30,31]. Twenty glass marbles were placed at one end of a regular rat cage half-covered with bedding for the test. Rats were placed at the opposite end of the cage from the marbles and then were allowed 30 min to bury the marbles. At the end of the trial, the number of marbles buried was counted for each. The individual conducting the assessment was blind to the treatment group for all animals.

#### 2.2.4. Novel Object Recognition (NOR)

For the NOR test, we utilized a rectangular open field chamber (26 cm × 26 cm × 45 cm), and a digital camera was used to record the behavior. The test consisted of three phases (5 min each): (1) a habituation phase, (2) a familiarization phase, and (3) a testing phase. For the habituation phase, rats were placed in an open field chamber and allowed to acclimate. In the familiarization phase, animals were exposed to two familiar objects similar in shape and size and were allowed to explore both objects freely for 5 min. Testing was repeated 24 h later, but one of the two objects from the first trial was replaced by a novel object. Behavior was video recorded, and time devoted to exploring each object was quantified using ANY-maze software, Version 7.3, (Wood Dale, IL, USA). Using these values, we calculated the percentage of novelty preference (%) using the formula: novel object exploration time/total (novel + familiar) object exploration time × 100% [32].

#### 2.2.5. Sucrose Preference Test (SPT)

SPT was utilized to study depression-like behaviors in each group. We utilized a two-bottle paradigm: one bottle containing tap water and another containing a 1% sucrose solution [10,33]. All trials were performed in the animal’s home cage. On day 1, rats were given two bottles containing normal water. On day 2, two bottles of sucrose solution were given. On the third day, one sucrose and one normal water bottle were placed in the cage to allow rats to learn to choose between the two. Rats were then water-restricted for 12 h before the formal test was carried out. The test was then performed on day 3 with one bottle of water and one bottle of sucrose solution. After the end of 12 h, the volume of each solution was tallied and the percentage of sucrose preference relative to total fluid intake was calculated [34] using the following formula: sucrose intake/(sucrose intake + normal water intake) × 100.

### 2.3. Tissue Collection

After the completion of all behavioral assays, rats were euthanized using CO_2_ asphyxiation followed by decapitation. Brains were quickly removed and dissected on ice. The prefrontal cortex and the hippocampus were manually isolated and flash-frozen in liquid nitrogen. Samples were stored at −80 °C until further processing.

### 2.4. Reverse Transcription-Quantitative Polymerase Chain Reaction (RT-qPCR)

Total RNA was extracted from the prefrontal cortex and hippocampus using TRIzol reagent (Ambion; Life Technologies, Carlsbad, CA, USA), followed by chloroform solubilization and ethanol precipitation [35,36], as performed previously. The reference sample for the study was dissected from rats treated with saline. The RNA concentration was determined for quantity using a Nanodrop^TM^ 2000 UV spectrophotometer (Thermo Scientific). Complementary DNA (cDNA) was generated from 2 μg of total RNA by the High-Capacity RNA-to-cDNA Kit (Applied Biosystem; Foster City, CA, USA) in a total volume of 20 μL. PCR was performed using a CFX Opus 96, Real-Time PCR System (Bio-Rad; Hercules, CA, USA), and Power Track^TM^ SYBR green master mix (Applied Biosystems, Waltham, MA, USA). PCR was performed with the following conditions: 3 min at 94 °C, followed by 40 cycles of a 3-step qPCR (94 °C for 10 s, 58–65 °C for 15 s, then 30 s at 72 °C). All primers were designed using (https://www.ncbi.nlm.nih.gov/tools/primer-blast/ (accessed on 13 February 2024) and purchased from (Thermo Fisher, USA). The sequences can be found in Table 1. Primer validation was conducted by determining optimal annealing temperatures and PCR efficiencies by using serial dilutions of cDNA. Melt curve analysis was performed to detect the presence of single amplicons without primer dimers. Duplicate samples were prepared for qPCR with 4.5 μL per well of test cDNA, including no RT (reverse transcriptase) control to assess the amount of DNA contamination present in an RNA preparation. The plate was centrifuged for 1 min at 650× *g* to position all reagents at the bottom of each well. Ct values obtained were taken for the analysis using the ∆∆Ct method [37].

### 2.5. Statistical Analysis

Weight changes and food intake were analyzed over 8 time points, including baseline and each week during the study, by repeated measures ANOVA. When a statistically significant overall group effect was found, multiple comparisons were made post hoc via Tukey’s test. In this analysis, the F-test (F-statistic) was used to assess the interaction between time points (within-subjects factor) and treatment (between-subjects factor). qPCR data were analyzed via one-way ANOVA, followed by Tukey’s post hoc test. One-way analysis of variance (ANOVA) is generally robust to deviations from normality, especially when group sizes are similar or identical, as in our experiment. A value of *p* < 0.05 was considered significant. Data were analyzed using GraphPad Prism Version 10 for Windows (Boston, MA, USA). To verify the sample size, we used G*Power software (Version 3.1, Franz Faul, University of Kiel, Kiel, Germany) to calculate the actual power achieved for all the analyses in the current manuscript. All analyses achieved a power of at least 0.80, which is the standard target when calculating sample size. All data are expressed as mean ± SEM.

## 3. Results

### 3.1. DOX-Treated Animals Had Reduced Body Weight and Food Consumption Compared to CBD

**Figure 2:**
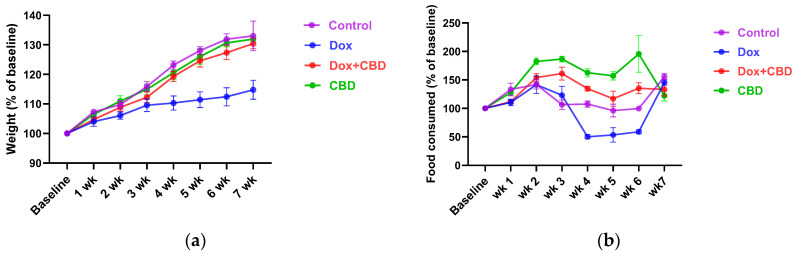
Effects of CBD and DOX on (**a**) body weight (*n* = 4/group), (**b**) food consumption (*n* = 4/group). Rats in all the treatment groups were monitored to study the effect on body weight and food intake over the entire study period. All the data points were collected at baseline and 1 week after treatment. Two-way repeated measures ANOVA with Tukey’s post hoc test was used to analyze the data and are presented as means ± SEM, DOX, doxorubicin; CBD, Cannabidiol.

Each rat’s body weight and food consumption were monitored over the DOX and CBD administration period. As shown in Figure 2a, animals in all groups gained weight throughout the study, as expected based on their increasing age. Repeated measures ANOVA revealed a significant time × treatment effect (F_21,84_ = 7.508, *p* < 0.001). DOX-treated animals tended to weigh significantly less than controls at week 4 (*p* = 0.03), week 5 (*p* = 0.02), and week 6 (*p* = 0.01).

Similarly, DOX-treated rats demonstrated reduced food intake (as a percentage of baseline food intake) over time (Figure 2b), (time × treatment interaction, F_21,84_ = 9.89, *p* < 0.0001). CBD-treated animals tended to consume more than the Control group rats at week 2 (*p* = 0.04), week 3 (*p* = 0.002), week 4 (*p* = 0.003), and week 5 (*p* = 0.02); (Figure 2b).

### 3.2. Elevated Plus Maze and Open Field Test

**Figure 3:**
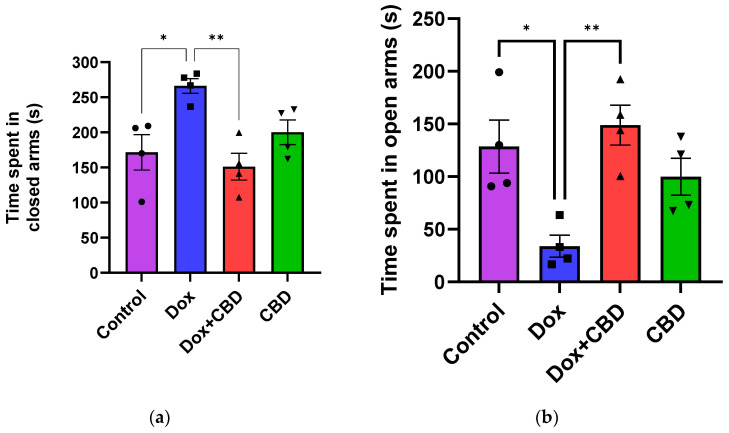
CBD treatment significantly alleviated anxiety-like behaviors induced by DOX treatment in EPM. (**a**) Time spent in closed arms. (**b**) Time spent in open arms. Data are presented as mean ± SEM (*n* = 4). * *p* ≤ 0.05, ** *p* ≤ 0.01, one-way ANOVA followed by Tukey’s post hoc test). DOX—doxorubicin; CBD—Cannabidiol.

An analysis of EPM data revealed that rats treated with DOX demonstrated a reduction in time spent in the open arms (F_3,12_ = 7.14; *p* = 0.005) and the number of entries in the open arm (F_3,12_ = 6.80; *p* = 0.006) compared to the normal Control group (Figure 3). However, the number of entries to the closed arm was not significantly different across the treatment groups (*p* > 0.05). Time spent (*p* < 0.04), distance traveled (*p* < 0.003), and the number of entries in the open arms (*p* < 0.02) were significantly increased in the (CBD + DOX)-treated animals when compared to the DOX-treated group.

**Figure 4:**
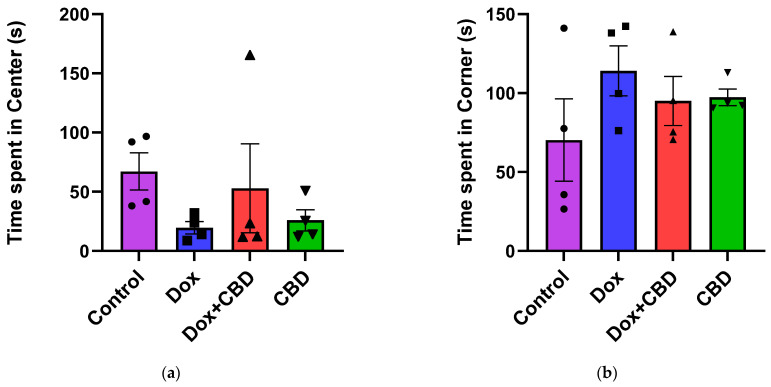
Effects of CBD and DOX treatment on Open Field Test. (**a**) Time spent in center. (**b**) Time spent in corner. Data are presented as mean ± SEM (*n* = 4; one-way ANOVA followed by Tukey’s post hoc test). DOX—doxorubicin; CBD—Cannabidiol.

For the OFT, there were no significant differences across groups for time spent in the center and time spent in the corner (Figure 4). Similarly, there were no significant differences for other parameters of the OFT, including the number of entries to the center, the number of entries to the corner, time spent in the walls, the number of entries to the walls, or freezing time.

### 3.3. Marble Burying Test

**Figure 5:**
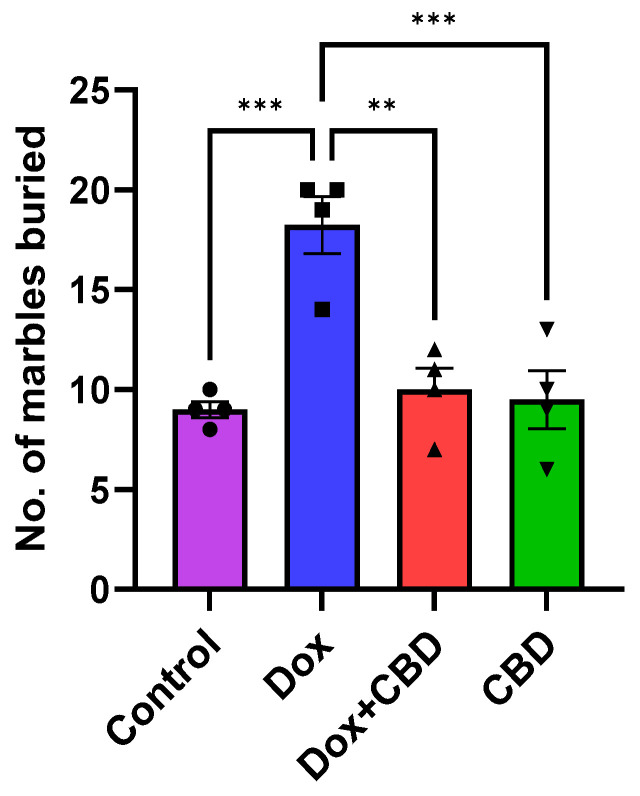
Anxiolytic effect of CBD against DOX treatment in Marble Burying Test (*n* = 4). Number of marbles buried was counted for each animal after 30 min of test session. Data are displayed as mean ± SEM (*n* = 4). ** *p* ≤ 0.01, *** *p* ≤ 0.001, (one-way ANOVA followed by Tukey’s post hoc test). DOX—doxorubicin; CBD—Cannabidiol. Some error bars may be too small to see on the chart.

As shown in Figure 5, DOX-treated animals buried a significantly greater number of marbles than the control (*p* < 0.001) and DOX + CBD (*p* < 0.001) groups. CBD treatment (*p* < 0.0001) shows a significant reduction in the number of buried marbles, suggesting a decrease in anxiety and repetitive behavior following CBD treatment. A strong main effect of treatment was noted in the ANOVA (F_3,12_ = 14.10, *p* < 0.001).

### 3.4. Novel Object Recognition

**Figure 6:**
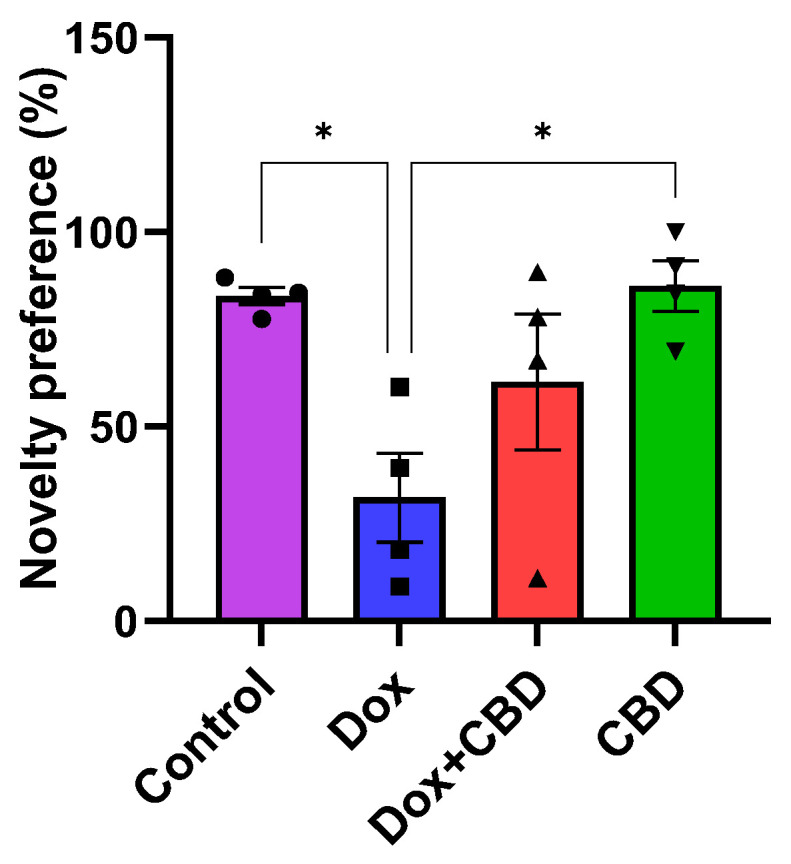
The percentage of preference for a novel object during the Novel Object Recognition test. The percentage of novel object preference was calculated as novel object exploration time/Total (novel + familiar) object exploration time × 100%. Data are presented as mean ± SEM (*n* = 4). * *p* ≤ 0.05 (one-way ANOVA followed by Tukey’s post hoc test). DOX—doxorubicin; CBD—Cannabidiol. Some error bars may be too small to see on the chart.

We investigated the ability of CBD to improve NOR memory in DOX-treated rats. Animals treated with DOX spent significantly less time exploring a novel object than the Control group (*p* = 0.02). However, rats treated with both DOX plus CBD displayed no significant preference in exploring a novel object compared to animals that were only given DOX (Figure 6). The treatment had a significant effect (F_3,12_ = 5.28, *p* = 0.0149). Furthermore, rats in the CBD-only group displayed a significantly greater preference for the novel object than the DOX-only (*p* = 0.01) group.

### 3.5. Effects of CBD on DOX-Induced Anhedonia in Sucrose Preference Test (SPT)

**Figure 7:**
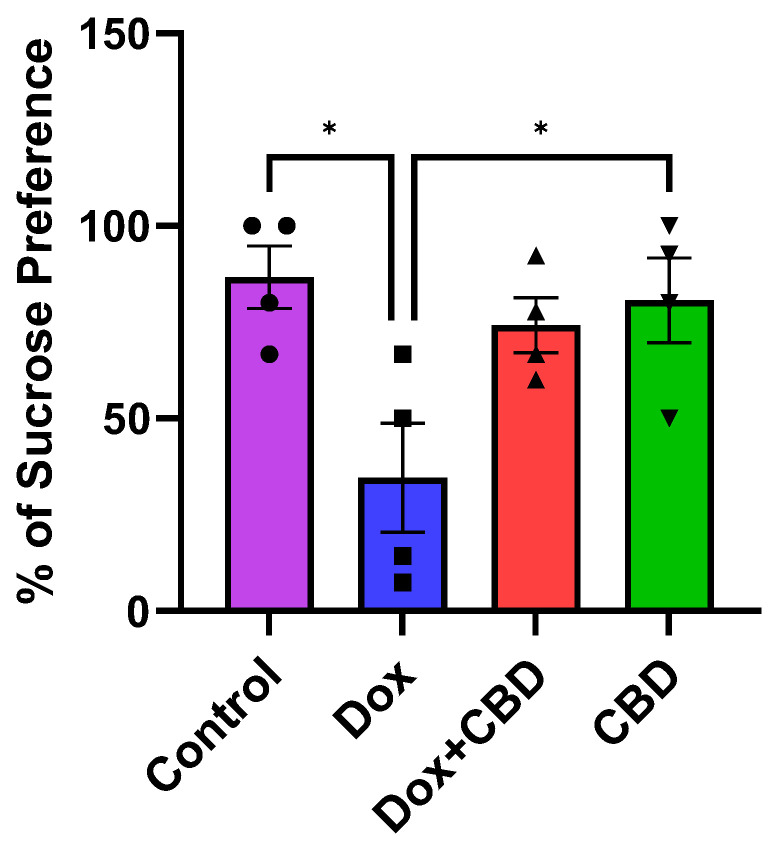
Effects of CBD on DOX-induced sucrose preference deficit. Percentage of sucrose preference (sucrose intake/total intake × 100). Data are displayed as mean ± SEM (*n* = 4). * *p* ≤ 0.05, one-way ANOVA followed by Tukey’s post hoc test). DOX—doxorubicin; CBD—Cannabidiol.

Although there was no significant time and treatment interaction (F_9,48_ = 0.69, *p* = 0.71) in the SPT, a significant treatment effect was found (F_3,48_ = 17.41, *p* < 0.0001). Multiple comparisons revealed that DOX-treated rats exhibited a significant reduction in sucrose preference as compared with the normal Control group (*p* < 0.001), indicating depression-like behaviors. However, rats treated with both DOX plus CBD displayed no significant sucrose preference compared to animals that were only given DOX (*p* > 0.05; Figure 7). Furthermore, rats in the CBD-only group displayed a significantly greater preference for sucrose water than the DOX-only (*p* = 0.01) group.

### 3.6. Expression of mRNA Following DOX in Prefrontal Cortex and Hippocampus

**Figure 8:**
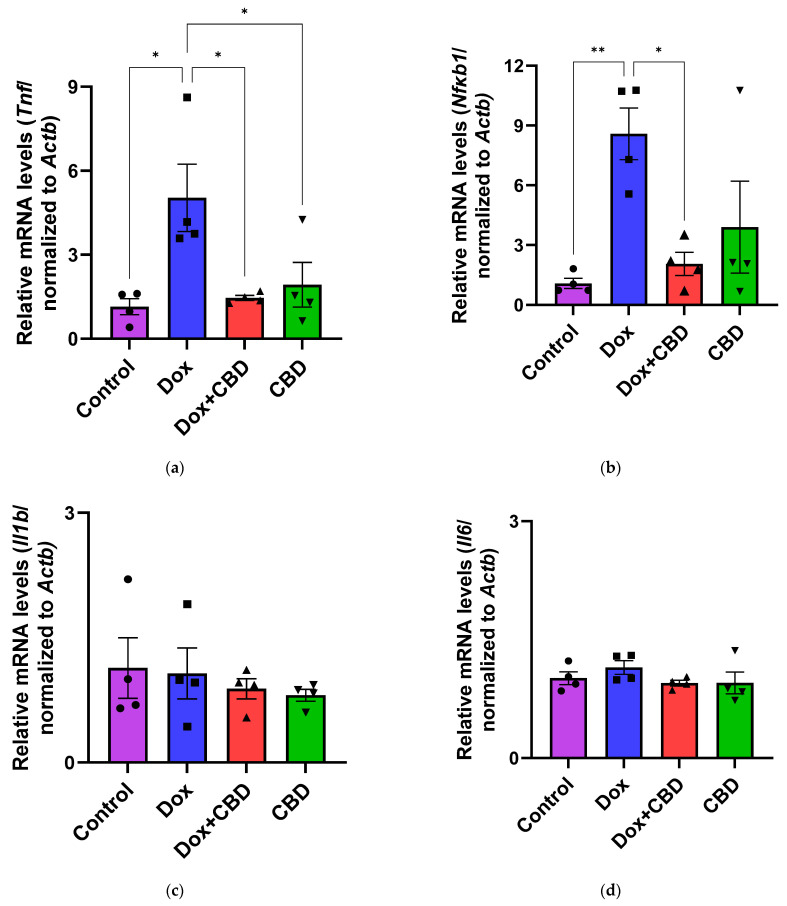

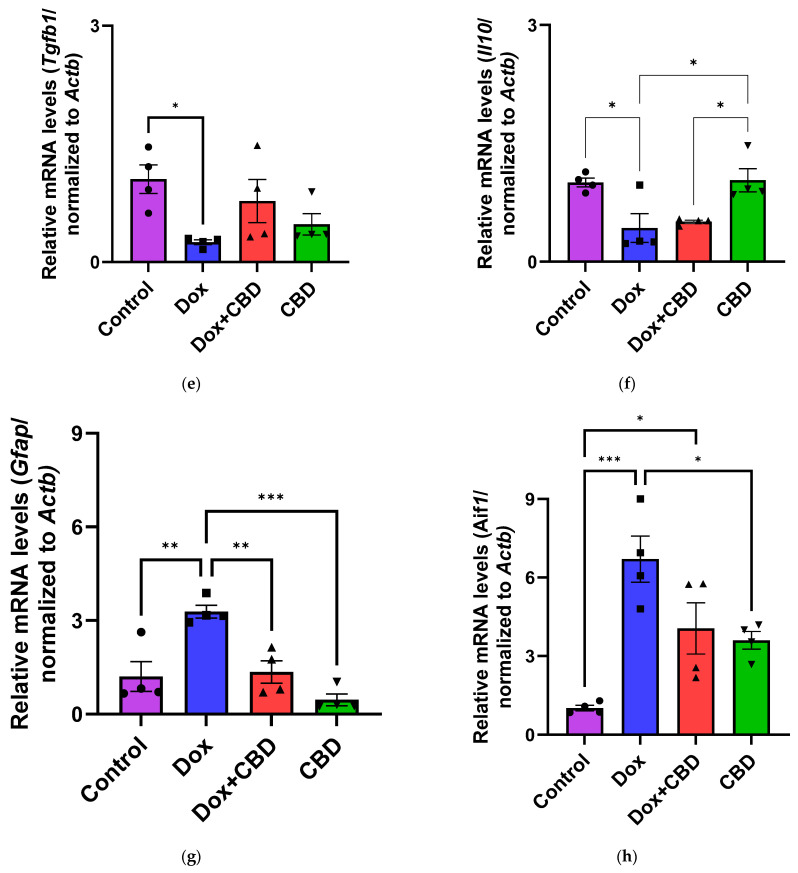
CBD suppressed neuroinflammatory response in the prefrontal cortex of DOX-injected rats. Quantification of gene expression by qPCR. Relative mRNA expressions of *Tnf* (**a**), *Nfkb1* (**b**), *Il1b* (**c**), *Il6* (**d**)*, Tgfb1* (**e**), *Il10* (**f**), *GFAP* (**g**), *Aif1* (**h**) were measured. Real-time PCR results were standardized against *Actb*, and data are presented as mean ± SEM. *n* = 4/group; *, **, ***: significant differences observed for *p* ≤ 0.05, *p* ≤ 0.01, and *p* ≤ 0.001, respectively (one-way ANOVA followed by Tukey’s post hoc test). DOX—doxorubicin; CBD—Cannabidiol; qPCR—real-time polymerase chain reaction; *Tnf*—tumor necrosis factor; *Nfkb1*—Nuclear factor kappa-light-chain-enhancer of activated B cells Subunit 1; *Il1b*—Interleukin beta 1; *Il6*—Interleukin 6; *Tgfb1*—Transforming growth factor beta 1; *Il10*—Interleukin 10; *Aif1*—Allograft inflammatory factor 1; *Gfap*—glial fibrillary acidic protein; *Actb*—beta actin. Some error bars may be too small to see on the chart.

**Figure 9:**
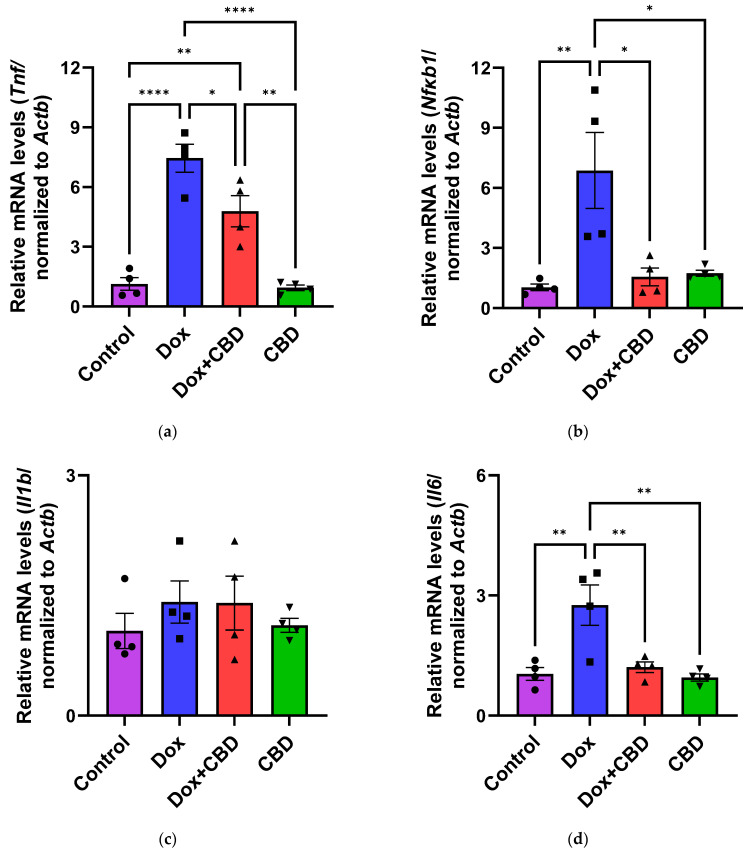

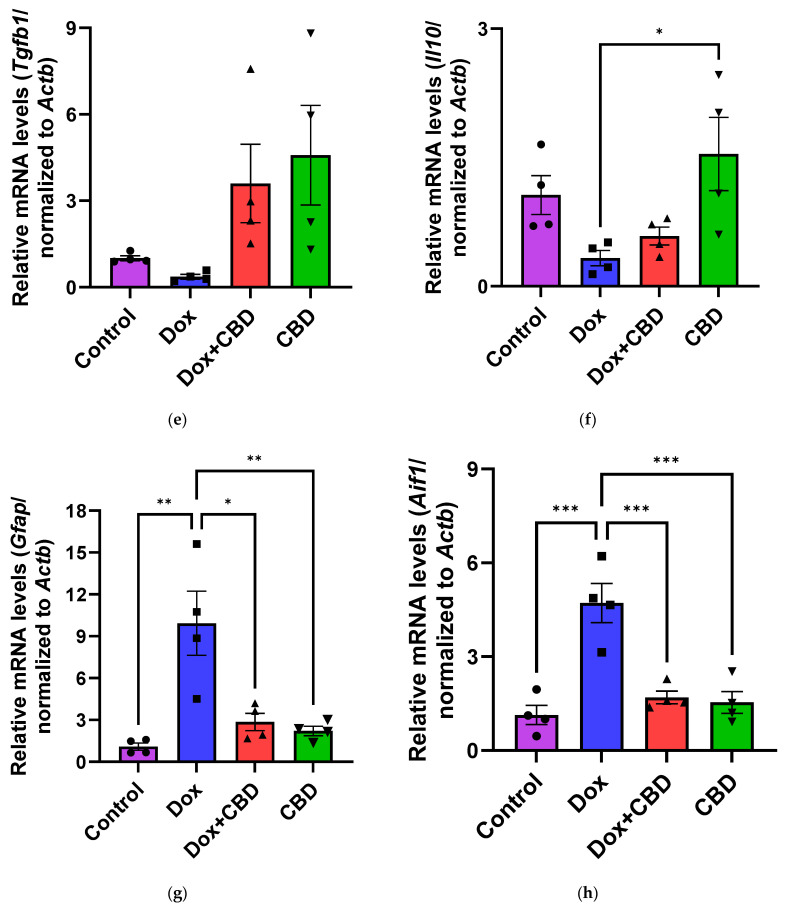
CBD suppressed neuroinflammatory response in the hippocampus of DOX-injected rats. Quantification of gene expression by qPCR. Relative mRNA expressions of *Tnf* (**a**), *Nfkb1* (**b**), *Il1b* (**c**), *Il6* (**d**)*, Tgfb1* (**e**), *Il10* (**f**), *GFAP* (**g**), *Aif1* (**h**) were measured. Real-time PCR results were standardized against *Actb*, and data are presented as mean ± SEM. *n* = 4/group; *, **, ***, ****: significant differences observed for *p* ≤ 0.05, *p* ≤ 0.01, *p* ≤ 0.001, *p* ≤ 0.0001 respectively (one-way ANOVA followed by Tukey’s post hoc test). DOX—doxorubicin; CBD—Cannabidiol; qPCR—real-time polymerase chain reaction; *Tnf*—tumor necrosis factor; *Nfkb1*—Nuclear factor kappa-light-chain-enhancer of activated B cells Subunit 1; *Il1b*—Interleukin beta 1; *Il6*—Interleukin 6; *Tgfb1*—Transforming growth factor beta 1; *Il10*—Interleukin 10; *Aif1*—Allograft inflammatory factor 1; *Gfap*—glial fibrillary acidic protein; *Actb*—beta actin. Some error bars may be too small to see on the chart.

The effects of CBD on mRNA expression of pro-inflammatory genes (*Tnf*, *Il1b*, *Il6*, and *Nfkb1*), anti-inflammatory genes (*Tgfb1* and *Il10*), as well as *Gfap* and *Aif1 (Iba1)* were determined using the tissue samples from the prefrontal cortex and hippocampus, respectively. qPCR analyses showed that DOX upregulated the expression of *Tnf* (*p* < 0.005) and *Nfkb* (*p* < 0.005) in the prefrontal cortex (Figure 8) and hippocampus (Figure 9) in comparison to the Control group. No significant differences existed between groups in *Il6* expression within the prefrontal cortex. However, elevated *Il6* expression persisted within the hippocampus after DOX treatment (Figure 9). No significant effect was observed in *Il1b* mRNA expression following DOX and CBD treatment. Rats in the DOX + CBD group showed lower mRNA levels of *Tnf* (*p* < 0.0001) and *Nfkb1* (*p* = 0.01) compared with those in the DOX-treated rats in both the brain regions studied (Figure 8 and Figure 9). In the prefrontal cortex, analyses of *Tgfb1* and *Il10* mRNA expression revealed a significant reduction in the DOX group compared to the Control group (*p* < 0.005), but no differences were observed in the hippocampus. CBD elevated the expression of *IL10* in both brain regions. Similarly, we also observed glial cell activation following DOX treatment, which was demonstrated by increased mRNA levels of *Gfap* (*p* = 0.002) and *Aif1* (*p* = 0.002) compared with control in both brain regions. Treatment with DOX + CBD produced a statistically significant decrease in *Gfap* mRNA expression in both brain regions (*p* < 0.01). Moreover, the administration of CBD alone also significantly decreased the relative mRNA expression of *Gfap* (*p* < 0.05) and *Aif1* (*p* < 0.05) in the prefrontal cortex (Figure 8) and hippocampus (Figure 9) in comparison to the DOX-treated group.

## 4. Discussion

Treatment-related cognitive deficits are one of the most common side effects of chemotherapy [1]. Previous studies in several rodent models have reported multiple possible mechanisms contributing to cognitive deficits following chemotherapy treatment with DOX, including inflammation, dysregulation of apoptosis, changes in neurotransmitter levels, glial cell interactions, neurogenesis inhibition, hormonal changes, alterations in synaptic plasticity, and long-term potentiation [3,4]. These mechanisms have been studied in parallel with anxiety and depressive behaviors for years [1,10].

Treatment with DOX frequently results in neurological side effects in animal models, including cognitive deficits [4,7,29,38,39]. Sameha et al. demonstrated that DOX-treated male Wistar rats showed reduced locomotor and exploratory behavior in Open Field and Elevated Plus Maze tasks [39]. In the current study, DOX-treated animals spent significantly less time exploring the open arms and more time in the closed arm of the Elevated Plus Maze, indicating anxious-like behavior. We did not find any difference in the Open Field Test between groups, which contrasts with the findings of Keeney et al., who reported an effect of doxorubicin on locomotor activity using the same test [40]. This discrepancy may be attributed to using different animal models and dosages. The anxiety-like behaviors observed in the Elevated Plus Maze were not confounded by locomotion in the Open Field Test, as there were no significant changes in rat behavior during their performance in the Open Field Test.

DOX-induced depression is often studied together with anxiety-like behaviors. In addition to problems in concentration, memory, and decision-making, individuals with depression experience changes in sleep patterns, changes in appetite, and thoughts of harming themselves [41,42]. Like anxiety, cancer patients may experience depressive symptoms before, during, and a long time after chemotherapy [43]. In addition to this, other studies [10,44] have shown an identical effect in different strains of rats that show depression-like behavior following DOX treatment, indicating that DOX administration can induce depression-like behaviors in rodents irrespective of species and dose administered. Consistent with previous studies, DOX-treated rats in our study exhibited depression-like behavior as manifested by a reduced sucrose preference index in the Sucrose Preference Test (Figure 7), which is indicative of anhedonia [44].

In patients with anxiety, 40% are resistant to the use of serotonin reuptake inhibitors (SSRIs) [45]. Given this substantial proportion of patients who may not benefit from SSRIs, it is important to explore alternative treatment options. In this context, investigating CBD as a potential treatment for anxiety becomes a logical and necessary step to address the needs of those who do not find relief with conventional therapies. CBD is highly recognized as a non-psychotomimetic component of *Cannabis sativa*, having a broad range of therapeutic potential [24,46,47]. While THC’s mechanism of action depends on CB1 and CB2 receptors, which are the two potent endocannabinoid system receptors, CBD is known to act independently of these receptors [17,48,49,50]. However, the precise mechanism of action for how CBD alters the behavioral changes induced by Chemo-brain has not been verified, to the best of our knowledge.

CBD has proven to be an effective medicinal therapy in several animal and human studies. Cunha et al. (1980) first reported that a CBD dose of 200 mg/day was effective in the treatment of seizures in humans [51]. A double-blinded, placebo-controlled trial was conducted among 120 children and young adults to study the efficacy of CBD against drug-resistant epilepsy in Dravet syndrome [52]. The study found that the use of CBD results in a greater reduction in convulsive seizure frequency than the control [52]. Unlike THC, CBD is non-euphoric in nature as it does not activate central CB1 receptors [53,54]. However, CBD has shown its potent antioxidant and anti-inflammatory role via its interaction with PPARy (Peroxisome Proliferator-Activated Receptor Gamma), TRPV1 (Transient Receptor Potential Vanilloid 1) channels, and 5-HT1A (5-Hydroxytryptamine Receptor 1A) receptors [53,55]. During these interactions, CBD is capable of activating the serotonin 1A receptor and blocking the activation of GPR55 (G-protein-Coupled Receptor 55, nucleoside transporter) and the TRP (Transient Receptor Potential) cation channel [56]. CBD can also interact with voltage-dependent anion-selective channel protein (VDAC1) [57]. Like THC, CBD is also highly lipid soluble. It gets easily distributed in the brain, adipose tissue, and several other organs [56]. The bioavailability of CBD depends upon the route of administration. For instance, CBD taken as oil or via oral route is believed to be most effective due to its lipophilic nature [56]. Over the past several decades, the unique pharmacological features of CBD and differential manipulations in dosing and the route of administration of CBD have provided a much better understanding of the potential of CBD to diminish psychotropic effects.

Interestingly, several other receptors, like 5HT1A, GPR55, and PPARγ, have been identified to be involved in the therapeutic actions of CBD [46,58]. Some studies have also mentioned that CBD modulates neuroprotection by increasing the levels of an endogenous cannabinoid called Anandamide (AEA) by inhibiting the anandamide hydrolysis pathway mediated by Fatty Acid Amide Hydrolase (FAAH) [46,59]. Benefits have been shown in mice with mild traumatic brain injuries, where oral cannabinoids improved social interactions and reduced aggressive behavior [17], and rats with neuropathic pain, where repeated CBD treatment prevented anxiety-like behavior [19,24].

Consistent with these studies, we detected lower anxiety symptoms in CBD-treated rats, as demonstrated by the less time spent in the closed arm of the Elevated Plus Maze and significantly more time exploring the open arm of the maze (Figure 3). Furthermore, CBD-treated animals buried a significantly lower number of marbles in the marble burying task, suggesting that the rats were less engaged in repetitive behavior after CBD treatment (Figure 5). This is an important finding because it shows that CBD may be effective against obsessive-compulsive disorder (OCD), which is one of the core symptoms of anxiety [60].

We also evaluated CBD for its potential to ameliorate impaired object recognition memory following DOX. In our rats, DOX administration resulted in impaired object recognition memory, as evidenced by the reduced novelty preference following DOX treatment in the Novel Object Recognition test. CBD-treated animals showed higher novelty preference than the DOX-treated group (*p* = 0.019), suggesting improved object recognition memory after CBD treatment (Figure 6). However, the DOX + CBD group showed no significant differences in object recognition compared to DOX. Oral treatment of CBD alone improves neuroprotection in our rats, but further studies are needed to prove the potential of CBD to improve object recognition memory following DOX treatment.

Immune response mediated by resident glial cells in the nervous system maintains neuronal health [61]. Any changes in these resident cells due to pathological conditions can lead to neuroinflammation, causing learning and memory dysfunction [62]. Neuroinflammation involves the activation of astroglia and microglial cells, leading to the release of proinflammatory cytokines. Increased levels of proinflammatory cytokines, including *Tnf* and *Il1b*, have been observed in patients following chemotherapy [40]. DOX-induced neuroinflammation has been widely studied in several animal models, including different strains of rats, such as Wistar, Sprague Dawley, and athymic nude rats [10,63,64,65]. Studies of the downstream effects of cannabinoid compounds in glial cells provide a basis for the study of the anti-inflammatory properties of CBD. For example, immune system cells express cannabinoid receptors, as evidenced by increased *Cnr1* mRNA expression [66] and increased *Cnr2* receptor expression in response to activated microglia and astrocytes [66]. Moreover, CBD could reduce the expression of cytokines, including *Tnf*, in a mouse model of hypoxic–ischemic brain damage, indicating its ability to reduce neuroinflammation [67].

Pro-inflammatory cytokines trigger cascades of pathways that may lead to glial cell activation, resulting in cognitive deficits [3,4]. Their expression by immune cells is highly capable of activating NF-κB transcriptional activity via inhibiting inhibitors of κB kinase and DNA binding domains of NF-κB [68]. We conducted gene expression analyses from the freshly dissected prefrontal cortex and hippocampal tissues of DOX- and CBD-treated rats to corroborate these findings. In our study, mRNA expression of the proinflammatory cytokines *Tnf*, *Nfkb1*, and *Il1b* were significantly increased after exposure to DOX alone but not in rats treated with DOX and CBD. Consistent with this, we found elevated hippocampal *Il6* mRNA levels after DOX treatment. Ongnok et al. also found a similar increase in mRNA expression of *Il6* following DOX in their model [11].

Conversely, we did not detect any significant differences in cortical mRNA levels after DOX treatment. DOX-induced activation of proinflammatory genes may occur in a brain-region-specific manner. CBD is capable of inhibiting iNOS (inducible Nitric Oxide Synthase) expression via the activation of the PPARγ pathway and increases anti-inflammatory cytokines [68]. In our study, the expression of anti-inflammatory genes, including *Il10* and *Tgfb1*, were all significantly decreased in DOX-challenged rats, while CBD treatment showed the opposite effects (Figure 8 and Figure 9).

CBD treatment reduced mRNA expression levels of *Aif1* and *Gfap*, indicating that CBD prevents glial cell activation. This implies that CBD might be a promising agent that can prevent neuroinflammation from the prolonged activation of microglia and astrocytes or potentially prevent cognitive deficits during chemotherapy treatment.

Our study revealed that neuroinflammation is vital in DOX-induced cognitive deficits, particularly hippocampal damage. Thus, our results substantially support previous reports of the neuroprotective benefits of CBD [17,28,44,46,48]. Neuroinflammation caused by DOX may contribute to the impairment of neurological functions. We found that the reduced expression of neuroinflammatory-associated mRNA corresponded with reductions in anxiety and depression-like behaviors in chemotherapy-treated animals given CBD. Consistent with the behavioral effects observed in the different treatment groups, our results showed increased mRNA expression of pro-inflammatory genes (*Tnf, Nfkb1,* and *Il1b*), reduced mRNA expression of anti-inflammatory genes (*Il10* and *Tgfb1*), as well as a marked increase in the gene expression of *Aif1* and *Gfap* in the prefrontal cortex as well as the hippocampus of DOX-treated animals, which was reduced by oral treatment with CBD.

Some limitations of our study should be addressed in the future to draw definitive conclusions about the efficacy of CBD in Chemo-brain. We recognize that a larger sample size may have enabled us to detect some more subtle differences in behavior or mRNA expression than our current data allow. However, our sample was sufficient to detect differences between treatment groups due to the effect sizes observed and yielded an overall power greater than or equal to 0.8 for all statistical tests performed.

Our preliminary study aimed to explore the effects of DOX specifically in a context relevant to breast cancer research. Therefore, we focused on female animals because DOX is most used in the treatment of triple-negative breast cancer [69]. Consequently, we did not assess sex differences in this initial phase. Other cancers where DOX is used include ovarian cancer, leukemia, and certain types of lymphoma, but breast cancer remains one of the most common applications. We acknowledge that future studies should consider including both male and female subjects to investigate any potential sex-based differences in response to DOX and CBD.

Additionally, DOX can influence appetite, as we show in Figure 2. This may impact the interpretation of our SPT results. Due to this potential confounder, follow-up studies are needed that incorporate additional measures to separate mood effects from appetite changes, such as including behavioral assays to evaluate mood or using food intake assessments alongside the SPT.

Our study does not account for tumor-induced animal models of cancer, as all experiments were performed using normal rats. Additionally, our study does not provide information regarding the expression of CBD receptors, which should be addressed in future experiments. Based on our findings that CBD treatment altered the neuroinflammatory mediators in normal rat hippocampi and prefrontal cortices, it would also be interesting to elucidate the neuroprotective effects of CBD under different dosing conditions.

## 5. Conclusions

In conclusion, this study demonstrated that the chronic, systemic administration of DOX impairs cognitive abilities in rats, increases anxiety and depression-like behaviors, and regulates the expression of genes involved in neuroinflammation. We found that CBD-treated rats had fewer anxiety and depression-like behaviors than rats treated with DOX alone. After finding robust alterations in the mRNA expression of neuroinflammatory genes, future experiments are needed to assess whether the observed change in mRNA expression affects protein expression. Although this study was a preliminary exploration with some limitations, it provides a foundation for further exploring CBD’s mechanism of neuroprotection from chemotherapy-induced cognitive deficits.

## Figures and Tables

**Table 1:** Sequences of primers used in real-time PCR analyses of mRNA expression.

Gene	Forward	Reverse
*Tnf*	ATGGGCTCCCTCTCATCAGT	GCTTGGTGGTTTGCTACGAC
*Il1b*	AGGAGAGACAAGCAACGACA	GTTTGGGATCCACACTCTCC
*Nfkb*	GAGACCTGGAGCAAGCCATT	GCTGCTCCTCTATGGGAACTTG
*Il6*	GCCCACCAGGAACGAAAGTC	TGGCTGGAAGTCTCTTGCGG
*Actb*	GGAGATTACTGCCCTGGCTCCTA	GACTCATCGTACTCCTGCTTGCTG
*Il10*	TTCCCTGGGAGAGAAGCTGA	GACACCTTTGTCTTGGAGCTTA
*Tgfb1*	CTGCTGACCCCCACTGATAC	AGCCCTGTATTCCGTCTCCT
*Gfap*	CGAAGAAAACCGCATCACCA	ATTTGGTGTCCAGGCTGGTT
*Aif1*	GCTATGAGCCAGAGCAAGGATT	CAAACTCCATGTACTTCGTCTTGA

## Data Availability

The raw data supporting the conclusions of this article will be made available by the authors upon request.
